# Are Hospital Patients Over-Fed?

**Published:** 1913-07-26

**Authors:** 


### PROBLEMS IN ADMINISTRATIVE MEDICINE.

(Carefully thought-out Contributions to this Section will be welcome.)

### Are Hospital Patients Over-fed?

FOR a long time critical observers of the system of medical education in Britain have noted that dietetics is a subject to which almost no attention is devoted by teachers and students in our medical schools. What knowledge the British practitioner, general or consultant, has of this vastly important section of therapeutics he has picked up for himself, as a rule, either by empirical practice on his patients or by post-graduate study of such text-books as are available. Of quite recent years the number of these text-books has increased and their quality much improved; it only requires some enthusiasm on the part of teachers, some recognition on the part of examining corporations, and dietetics might receive sufficient attention to remove entirely the reproach of neglecting it which at present certainly does still attach to British medicine.

### PRACTICAL DIETETICS.

When some years ago scientific experimental study of metabolism was seriously taken up, there was a tendency to classify foods mainly by the number of heat or energy units which they yielded when oxidised by the vital processes in the tissues; and some aftermath of this tendency is still to be found in text-books of dietetics. The most modern authorities, however, have now fully recognised the importance of other factors in the selection of foods, such as assimilability, palatability, provocation of appetite, various physical and psychical effects which depend on the personal equation of the consumer. The results disclosed a few years ago by THE HOSPITAL's Commission of investigation into the action of wines, beers, and spirits are a forcible illustration of this aspect of the problems of food and drink in their relation to the nutrition of human beings. This and similar lessons with regard to many other foodstuffs have had their effect, and the best books on diet to-day are far more practical in scope and outlook than those of, say, ten years ago. Among them we know of none better than that by Dr. Chalmers Watson,* a second edition of which has now appeared some two and a half years only after its original issue. This work is a veritable storehouse of information upon the many aspects of practical dietetics, and it is especially valuable to institutional authorities because of its pertinent criticisms of ward dietaries. As Dr. Watson points out, one of the consequences of the failure to instruct the twentieth-century medical student in dietetics is that the dietary arrangements for hospital patients are left by the junior medical officers to an undue extent in the hands of the nursing staff. Where an experienced matron or housekeeper keeps the kitchen department thoroughly efficient, and where her ward sisters also take a lively interest in the feeding of the patients, this arrangement may work well enough. But where there is the slightest default in either of these directions disorganisation of the dietary part of the hospital treatment is inevitable, with corresponding disadvantages to the sick inmates.

### OVER-FEEDING IN HOSPITALS.

In many institutions, says Dr. Watson, the official dietary leaves little or nothing to be desired, yet the actual dietary is in a manner faulty. As an illustration of this thesis he gives in parallel columns a comparison of the official and the actual dietary of a large infirmary, and he contends that the difference leads to actual over-feeding of the patients and is detrimental to their progress, except in those cases where chronic starvation has preceded admission to the institution. Thus the official full dietary consists of: *Breakfast* (8 A.M.), coffee or tea; butter, ½ oz.; bread, 6 oz.; porridge, and ½ pint of milk, if desired. *Dinner* (1.30 P.M.), meat (roast or boiled), 8 oz.; vegetables, 12 oz.; bread, 4 oz.; soup (and pudding once a week in lieu of soup). *Tea* (4.30 P.M.), bread, 6 oz.; butter, ½ oz.; tea. Whereas the actual dietary contains not only these ingredients, frequently in quantity greater than that laid down, but also a *morning snack* at 5 A.M. consisting of bread and butter, with milk or beef-tea; a *lunch* of much the same nature; an egg with the afternoon meal; and a *supper* also, similar to the morning snack.

Too much food, says this author, is often given to hospital patients by over-zealous nurses who are anxious to hasten convalescence. This indictment of hospital nursing is rather sweeping, and we doubt if it can be sustained as a general proposition, though no doubt it could be proved in individual instances. Every practising medical man knows how hard it is to persuade the relatives and friends of sick people that a few days on light diet will not result in dangerous inanition, and to prevent them from overstraining the impaired digestive functions of some one who only requires a few days' complete rest, bodily and stomachic, to recover the capacity for resuming normal functions. In the case of the private practitioner, experience soon teaches him the absolute necessity of drawing up a diet table which shall specify not only the times of meals and their respective menus, but also the quantity of each article which shall be offered for consumption. If in hospital the medical officers, from ignorance or slackness or inexperience, neglect to look after this part of the patient's treatment, it will naturally fall into the hands of the sister and nurses; and, although in most cases no harm may ensue, it is this dereliction of responsibility that renders possible the facts of which Dr. Chalmers Watson complains.

* *Food and Feeding in Health and Disease.* By Chalmers Watson, M.D., F.R.C.P.E. (Edinburgh: Oliver and Boyd. Second Edition. Pp. 638. Price 10s. 6d. net.)

A Scheme of Hospital Diets.

We have quoted some of his comments on hospital dietaries in being; but it should not be supposed that Dr. Watson confines himself to destructive criticism. Throughout his book he is always essentially practical, and he concludes his chapter on hospital dietaries with a classification of them which institutional managers and physicians would do well to study carefully. The diets are six in number, rather more than the usual number of stock diets in English institutions.

First comes Milk Diet, consisting of four to six pints of milk daily, best divided into six meals. Alternative forms of this food are admissible, such as whey, skimmed milk, and koumiss. The second is Fluid Diet, consisting of milk, beef-tea, chicken broth, mutton broth, and egg albumin. Six meals daily are given as with Milk Diet, and the milk or beef-tea may be thickened with one of the predigested invalid foods.

As a specimen of Light or Convalescent Diet, the following scheme is proposed. *Breakfast*: milk flavoured with tea or coffee; bread and butter, or toast and butter; gruel, or hominy, or lightly cooked egg, or steamed fish. *Dinner*: soup; meat or chicken broth, thickened with barley or rice; dry bread or toast; fish or chicken; pudding made from invalid food, or stewed fruit. *Tea*: milk flavoured with tea; rusk, or sponge biscuit. *Supper*: a cupful of gruel, or malted gruel, or invalid food, or chicken broth, with a slice of toast.

Lacto-Vegetarian or Farinaceous Diet is one from which animal foods, with the exception of milk and butter, are excluded. Protein or Nitrogenous Diet is one from which, on the other hand, starchy and saccharine foods are largely excluded; it consists mainly of meats, fish, eggs, and selected vegetables in small quantities. Last of all comes Ordinary or Full or House Diet, a typical and adequate scheme of which has been given above. In connection with all these schedules of diet, the author gives excellent recipes for preventing satiety, creating appetite, and ensuring both economy of foodstuffs and that variety of food which is so important in catering for the capricious palates of sick and convalescent patients. Besides the regular diets, necessity occasionally compels the administration of nutriment per rectum, as an enema, or by means of a stomach tube passed by the mouth or nose. Full instructions as to the best ingredients, quantities, and methods of preparation of these diets are given in Dr. Watson's book, to which reference should be made direct for details. Those who consult this admirable book will find it a singularly complete, accurate, and lucid summary of the subject with which it deals, and, as medical books go nowadays, it is published at a very moderate and entirely reasonable price, considering what a lot it contains.

